# Role of Osteopontin in Systemic Lupus Erythematosus

**DOI:** 10.1007/s00005-014-0294-x

**Published:** 2014-06-11

**Authors:** Beata Kaleta

**Affiliations:** Department of Clinical Immunology, Transplantation Institute, Medical University of Warsaw, Nowogrodzka 59, 02-006 Warsaw, Poland

**Keywords:** Osteopontin, Systemic lupus erythematosus, Gene, Polymorphism

## Abstract

Systemic lupus erythematosus (SLE) is a multisystemic disease, caused by a variety of factors, which lead to immunological abnormalities. Osteopontin (OPN) is a pleiotropic protein, important in bone remodeling and immune system signaling. OPN, produced by various cells, including immune cells, plays a key role in regulating T-helper 1/T-helper 2 balance, stimulating B lymphocytes to produce antibodies, regulating macrophages, neutrophils and inducing dendritic cells. OPN expression is influenced by genetic polymorphisms of its promoter, hormones and cytokines. Over expression of OPN has been associated with the pathogenesis of immune-mediated diseases. OPN has been implicated in the development of murine model of lupus and in humans with SLE. In this review, I will present current state of research on the role of OPN and *OPN* gene polymorphisms in pathogenesis and clinical course of SLE. A better understanding of the role of OPN in SLE will contribute to more precise diagnosis and treatment of the disease.

## Introduction

Systemic lupus erythematosus (SLE) is a chronic multisystemic disease of autoimmune origin, characterized by involvement of multiple organs (skin, lungs, heart, joints, blood vessels, liver, kidneys, hematologic and nervous systems). SLE is caused by environmental, hormonal and genetic factors which lead to immunological abnormalities (Kotzin 1996; Mills 1994). Deregulation of B and T lymphocytes activation leads to production of autoantibodies and abnormalities in cytokine expression. Autoantibodies [against double-stranded DNA (ds-DNA), SS-A (anti-Ro), SS-B (anti-La), anti-Sm, anti-RNP] form complexes with antigens, which are deposited in organs, cause inflammation and tissue damage (Giles and Boackle 2013; Lindop et al. 2012; Marks and Tullus 2012; Rekvig et al. 2012). Deregulation in cytokine expression followed by inflammatory processes is also a cause of tissue injury. It was demonstrated that interleukin (IL)-6, B lymphocytes stimulators, IL-17, IL-18 and interferon (IFN)-α have the pathogenic role in SLE (Yap and Lai 2013).

Osteopontin (OPN), also known as early T lymphocyte activation-1 or secreted phosphoprotein 1, is a member of the small integrin-binding ligand N-linked glycoprotein (SIBLING) family proteins (Fisher et al. 2001; Ramaiah and Rittling 2008; Rangaswami et al. 2006). OPN for the first time was identified in 1986 as a major sialoprotein of bone, linking bone cells and hydroxyapatite, the main inorganic constituent of human bone tissue (Oldberg et al. 1986). OPN is produced by various cell types such as immune cells: B cells and T cells, natural killer (NK)T cells, NK cells, macrophages, neutrophils, dendritic cells (DCs), bone cells (osteoblasts and osteocytes), breast epithelial cells or neurons. OPN can be found in many organs such as bone, lung, liver, brain, joints, adipose tissue and body fluids: blood, urine, bile and semen (Denhardt and Noda 1998; Ramaiah and Rittling 2008; Uede 2011). OPN has been found to be an effective biomarker for a number of cancers (Afify et al. 2009; Ramaiah and Rittling 2008) and immune-mediated diseases, including multiple sclerosis (Carecchio and Comi 2011; Harris and Sadiq 2009; Murugaiyan et al. 2008), SLE (Kariuki et al. 2009; Kariuki and Niewold 2010; Murugaiyan et al. 2008; Zandman-Goddard and Shoenfeld 2003), rheumatoid arthritis (Cantor 1995; Gravallese 2003; Murugaiyan et al. 2008), atherosclerosis (Cho et al. 2009; Scatena et al. 2007), cardiovascular disease (Singh et al. 2007), inflammatory bowel disease (Glas et al. 2011; Mishima et al. 2007), asthma (Frenzel and Weiss 2011; Konno et al. 2011) and liver diseases (Cao et al. 2012; Ramaiah and Rittling 2008; Yilmaz 2012).

## *OPN* Gene: Structure and Polymorphism

Human *OPN* gene is mapped on the long arm of chromosome 4 (4q21–4q25). It is composed of seven exons. The first exon is untranslated. Exons 2–7 contain coding sequences. *OPN* gene promoter includes several types of regulatory sequences: the TATA box, GC box, reverse CCAAT cassette and several transcription factor-binding sequences: PEA3, E2A, AP1 and Ets (Sodek et al. 2000). Moreover, it was shown that *OPN* gene expression is affected by a number of cytokines (e.g., IL-1β, IL-6, tumor necrosis factor (TNF)-α, IFN-γ), hormones (vitamin D, estrogen, angiotensin II, glucocorticoids), platelet-derived growth factor and oxidized low-density lipoprotein (Denhardt and Noda 1998). In addition, the expression of OPN is influenced by genetic polymorphisms of its promoter (Chiu et al. 2010; Jiang et al. 2013). *OPN* gene is highly polymorphic. Several polymorphisms in the human *OPN* gene have been identified: in the 5′ flanking region, in exons and introns and in the 3′ untranslated region (Giacopelli et al. 2003; Iwasaki et al. 2001; Mochida et al. 2004).

## Structure, Metabolism and Function of OPN

Osteopontin is a pleiotropic protein and its functions are linked to various physiological functions and pathological conditions. OPN interacts with cells via two binding domains. Through the adhesive RGD motif (arginine–glycine–aspartate domain), OPN interacts with αvβ1, αvβ3, αvβ5, αvβ6, α8β1 and α5β1 integrins (Denda et al. 1998; Hu et al. 1995; Yokosaki et al. 2005). Moreover, OPN contains a SVVYGLR domain that mediates interactions with α9β1, α4β1 and α4β7 integrins (Green et al. 2001; Ito et al. 2009). OPN signaling via integrins modulates the phosphorylation of kinases which are involved in NF-κB activation and regulation of cytokines production (Urtasun et al. 2012). Osteopontin contains an aspartate-rich region near the C-terminal sequence. This domain is exposed as a result of proteolysis by thrombin and is able to interact with the CD44 receptors. It enables T cell chemotaxis and fibroblast adhesion. CD44 ligation with OPN leads to reduction in IL-10 gene expression in macrophages (Weber et al. 1996).

Osteopontin function is highly modified by post-translational modifications, including phosphorylation, O-linked glycosylation, sialylation and tyrosine sulfation. They are necessary for OPN regulation of mineralization, bone resorption and migration of cancer cells (Al-Shami et al. 2005; Gericke et al. 2005; Kazanecki et al. 2007; Razzouk et al. 2002).

OPN, secreted by osteoblasts, osteoclasts and osteocytes, is important in mineralization and bone resorption (Denhardt and Guo 1993; Denhardt and Noda 1998). Recently, this protein was found to be relevant in regulation of immunity and inflammation, angiogenesis, oncogenesis, cancer progression and apoptosis (Cantor 1995; Cao et al. 2012; Denhardt and Guo 1993; Murugaiyan et al. 2008). Due to the fact that OPN is expressed by many different cell types of the immune system, is up-regulated in response to injury and inflammation and regulates immunological response, it may be classified as a cytokine (Brown 2012; Denhardt and Guo 1993; Heilmann et al. 2009; Koh et al. 2007; Wang and Denhardt 2008). Osteopontin is highly expressed by macrophages and regulates their migration, activation, capacity for phagocytosis and nitric oxide production (Ashkar et al. 2000; Brown 2012; Wang and Denhardt 2008; Weber et al. 1996). It has been demonstrated that OPN is a chemoattractant for neutrophils (Koh et al. 2007; Wang and Denhardt 2008) and induces DCs maturation. OPN promotes activation of T lymphocytes, and regulates the T-helper 1 (Th1)/Th2 balance. Via interaction with αvβ3 integrin, OPN up-regulates IL-12. Through CD44 receptor, OPN downregulates IL-10 (Ashkar et al. 2000). Recent findings revealed that non-secreted form of OPN enhances IFN-α expression through the IFN regulatory factor 7 activation upon Toll-like receptor (TLR)9 stimulation in plasmacytoid DCs (pDC) (Shinohara et al. 2006). Moreover, OPN activates and stimulates antibodies production by B lymphocytes (Ashkar et al. 2000; Iizuka et al. 1998; Lampe et al. 1991). In addition, some studies suggested that OPN enhances IL-17 producing Th17 cell responses by inhibiting the production of IL-27 and IL-17 inhibitor produced by pDC (Murugaiyan et al. 2008; Shinohara et al. 2008).

## Role of OPN in SLE

### Studies Performed in Murine Models of SLE

OPN was reported to be highly expressed in the MLR/lpr mouse (commonly used model of the human SLE) (Iizuka et al. 1998; Lampe et al. 1991; Miyazaki et al. 2005; Weber and Cantor 2001; Wüthrich et al. 1998). In mice, CD4^−^/CD8^−^ T cells expressed high levels of OPN. Overexpression of OPN in murine model of SLE induces B cell activation and IgG and IgM production, elevated autoantibodies (including anti-ds-DNA) levels and enhanced cytokine expression (TNF-α, IFN-γ and IL-1β) (Iizuka et al. 1998; Miyazaki et al. 2005; Weber and Cantor 2001; Wüthrich et al. 1998). Overproduction of these cytokines has been shown to be involved in the pathogenesis of SLE (Yap and Lai 2013). The biological importance of OPN in IFN-α responses was emphasized by the finding that in the absence of OPN in mice, IFN-α production by pDC through TLR9 pathway was impaired (Shinohara et al. 2006). Moreover, it has been found that elevated circulating levels of OPN are associated with renal damage in the MLR/lpr mice (Miyazaki et al. 2005; Ophascharoensuk et al. 1999; Wüthrich et al. 1998).

### Studies Performed in Humans

In the literature, there are reports suggesting that OPN participates in the pathogenesis of some autoimmune diseases (Cantor 1995; Cho et al. 2009; Denhardt and Guo 1993; Gravallese 2003; Murugaiyan et al. 2008; Ramaiah and Rittling 2008; Scatena et al. 2007; Sodek et al. 2000; Uede 2011).

In 1989, first report was published to connect OPN to immunity (Patarca et al. 1989). In 1995, Katagiri and colleagues for the first time evaluated whether elevated OPN level can be detected in patients with autoimmune diseases (Katagiri et al. 1995). These authors observed that serum OPN level is elevated in SLE and RA patients compared with healthy donors. Moreover, they described two forms of OPN: large (64,000 Da) and small (32,000 Da). The small form of OPN may be derived from the large since it can be cleaved by thrombin. This thrombin-cleaved OPN exposes an epitope for integrin receptors: α4β1, α9β1 and α9β4 (Green et al. 2001; Ito et al. 2009). Li et al. (1999) and Lou et al. (2006) likewise demonstrated that plasma OPN concentration is elevated in SLE patients. In another study, Wong et al. (2005) measured the plasma concentration of OPN in 54 Chinese SLE patients with (renal disease in SLE: RSLE) and without (SLE) renal impairment. They demonstrated that OPN concentrations were significantly higher in SLE and RSLE patients than in controls (*p* < 0.001) and correlated positively and significantly with SLE disease activity index (SLEDAI) (*r* = 0.308, *p* = 0.023; Wong et al. 2005). Moreover, in RSLE patients, OPN concentration showed a positive correlation with the pro-inflammatory cytokine IL-18 level (*r* = 0.404, *p* = 0.037). This cytokine exerts a variety of effects on DCs, T lymphocytes and NK cells, and is an inducer of IFN-α to promote Th1 differentiation (Yap and Lai 2013). Wong et al. (2005) suggested that in combination with the inflammatory activities of IL-18, OPN can enhance the Th1-mediated inflammatory process in the exacerbation of SLE and RSLE. Similar finding was observed by Afify et al. (2009) in children with SLE (40 patients and 30 matched controls). The study showed that OPN concentrations were significantly higher in SLE patients with and without renal disease than in the controls (*p* = 0.000 and *p* = 0.002, respectively). Moreover, plasma OPN level correlated positively with disease activity index in both groups (*r* = 0.34, *p* = 0.04). In patients with renal disease, there was a positive correlation between IL-18 and OPN concentration (*r* = 0.48, *p* = 0.004). Rullo et al. (2013) hypothesized that increased circulating plasma OPN levels may be associated with organ damage in pediatric SLE (*n* = 42) and adult SLE (*n* = 23). Plasma OPN concentrations were significantly higher in both groups than in controls (*p* = 0.03 and *p* = 0.02, respectively). In addition, Rullo et al. (2013) showed that increased OPN is associated with higher titer of antibodies to ds-DNA (*p* = 0.001) and elevated IFN-α (*p* = 0.02), which were shown to be important in pathogenesis and clinical manifestations of SLE (Bengtsson et al. 2000). Moreover, Rullo et al. (2013) showed a positive correlation between OPN and SLEDAI in six months.

These results (summarized in Table 1) suggest that the production of OPN is associated with SLE development and may serve as a potential marker of SLE activity and organ damage.

**Table 1:** Main studies about osteopontin (OPN) role in human SLE

References	Study population	Relevant results
Katagiri et al. (1995)	10 SLE patients	Serum OPN level were elevated in SLE patients but not in healthy donors
		Two forms of OPN: large and small were detected. The small form may be derived from the large since it can be cleaved by thrombin
Wong et al. (2005)	54 SLE patients	Plasma OPN concentrations were significantly increased in SLE and RSLE patients (*p* < 0.001) and the elevation correlated positively with SLEDAI (*r* = 0.308, *p* = 0.023)
		In RSLE patients, OPN concentration showed a positive correlation with IL-18 level (*r* = 0.404, *p* = 0.037)
Lou et al. (2006)	38 SLE patients	Plasma OPN concentrations were significantly higher in SLE patients than in controls (*p* < 0.001) and showed a significant positive correlation with SLEDAI (*r* = 0.93, *p* < 0.001)
Afify et al. (2009)	40 pSLE patients	Plasma OPN concentrations were significantly higher in SLE and RSLE patients than in controls (*p* = 0.002 and *p* = 0.000) and correlated positively with SLEDAI (*r* = 0.34, *p* = 0.04)
		In patients with renal disease, there was a positive correlation between IL-18 and OPN concentration (*r* = 0.48, *p* = 0.004)
Rullo et al. (2013)	42 pSLE patients and 23 SLE adult patients	Plasma OPN concentrations were significantly higher in pSLE and adult SLE patients than in controls (*p* = 0.03 and *p* = 0.02) and the elevation correlated positively with six-month SLEDAI in both groups (*r* = 0.51 and 0.51, *p* = 0.001 and *p* = 0.01)
		Increased OPN at baseline was associated with antibodies to ds-DNA (*p* = 0.001) and elevated IFN-α (*p* = 0.02)

*OPN* osteopontin, *SLE* systemic lupus erythematosus, *pSLE* pediatric SLE, *RSLE* renal disease in SLE, *SLEDAI* SLE disease activity index

## Role of *OPN* Gene Polymorphism in SLE

### Studies Performed in Murine Models of SLE

Polymorphic OPN alleles have been implicated in the mouse model of lupus in the study of Miyazaki et al. (2005). These authors reported that *OPN* gene polymorphism induces enhanced expression of immunoglobulins: IgG3, Ig2a and IgM and cytokines: IFN-γ, TNF-α, IL-1β which play an important role in lupus mice models and in human SLE. It was shown that type I IFN signaling pathway is essential for up-regulation of TLR7 and TLR9 receptors in B lymphocytes and activation of B lymphocytes through these receptors to produce lupus-specific autoantibodies (Thibault et al. 2008). The role of TNF-α in lupus mice models is controversial. In some mouse models, the deficiency of TNF-α provoked autoimmunity (Kontoyiannis and Kollias 2000). In contrast, TNF-α concentration was elevated in sera and renal tissue of MLR/lpr lupus mice and the levels of this cytokine correlated with the severity of kidney disease (Yokoyama et al. 1995). Overproduction of IL-1β has been shown to be involved in the pathogenesis of SLE. Mice deficient in IL-1β developed lower levels of anti-ds-DNA antibodies (Voronov et al. 2006).

### Studies Performed in Humans

A number of studies demonstrated that increased plasma concentration, as a result of *OPN* gene polymorphism and increased protein expression, was associated with SLE susceptibility and/or clinical manifestations of the disease in humans. Study of Forton et al. (2002), including 81 Caucasoid SLE patients and Caucasoid 79 controls, showed that polymorphic T allele of the silent polymorphism 707 C>T (rs1126616) is associated with opportunistic infections and renal insufficiency (*p* = 0.0598 and *p* = 0.0431, respectively) but is protective for avascular necrosis (*p* = 0.0382). There was no association with cutaneous lupus, gastrointestinal lupus and specific laboratory features (Forton et al. 2002). This was the first demonstration of a phenotypic association with an *OPN* gene polymorphism but the authors admitted that the number of patients participating in the study was too low. In a study of 394 Italian SLE patients and 479 matched controls, a total of 13 single nucleotide polymorphisms (SNPs) in *OPN* gene were identified (six in the 5′ flanking region, one in intron 3, three in exons 6, 7 and 3 in the 3′-UTR; Table 2) (D’Alfonso et al. 2005). Five of these SNPs were newly identified. Two polymorphisms: −156G>GG (rs7687316) and +1239A>C (rs9138) were significantly associated with SLE. The −156G and +1239C alleles were more frequent in SLE patients than in the control group (*p* = 0.006 and *p* = 0.00094, respectively). In addition, significant association was seen between lymphadenopathy and −156 genotypes (*p* = 0.0011). Significantly increased OPN serum level was detected in healthy individuals carrying +1239C (*p* = 0.002). The authors suggested that −156G>GG and −1239A>C polymorphisms predispose to high production of OPN and susceptibility to SLE.

**Table 2:** Main studies of association between *OPN* gene polymorphisms and human SLE

References	Population	Cases/controls	Polymorphism	Relevant results
Forton et al. (2002)	American Caucasian	81/79	707C>T (rs1126616)	Polymorphic T allele was associated with renal insufficiency (*p* = 0.0431) and opportunistic infections (*p* = 0.0598), but protective for avascular necrosis (*p* = 0.0382)
D’Alfonso et al. (2005)	Italian	394/479	−156G>GG (rs7687316); +1239A>C (rs9138); −1748A>G (rs2728127); −1282A>G; −616G>T (rs2853744); −707C>T (rs11226616); +282T>C (rs4754); +1038A>G (rs1126772); −443T>C; −66T>G; IV3−42A>C; +351T>C; +1158A>C	Alleles −156G and +1239C were significantly increased in the SLE patients compared with controls (*p* = 0.006 and *p* = 0.00094, respectively). Significant association was seen between lymphadenopathy and −156 genotypes (*p* = 0.0011). Significantly increased OPN serum level was detected in healthy individuals carrying +1239C (*p* = 0.002)
Xu et al. (2007a, b)	Han Chinese	158/180	9250C>T	The frequency of TT genotype was significant lower in than in controls (*p* = 0.001). The frequency of TC genotype was significantly higher in patients than in controls (*p* = 0.088). The TT genotype was lower in patients with lupus nephritis (*p* < 0.05)
Han et al. (2008)	European-American (EA) and African-American (AA)	1,141/2,009 (707 EA/1309 and 434 AA/700)	32 SNPs in *OPN* gene	rs1126616T and rs9138C alleles were associated with higher risk of SLE in EA and AA combined males (*p* = 0.0005, OR 1.73, 95 % CI 1.28–2.33) but not in females. Significant gender–gender interactions in rs1126772 and rs9138 were detected (*p* = 0.001 and *p* = 0.0006, respectively). Haplotype analysis identified rs1126616T-rs1126772A-rs9138C which demonstrated association with SLE in general (*p* = 0.02, OR 1.30, 95 % CI 1.08–1.57), especially in males
Trivedi et al. (2011)	African-American (AA), European-American (EA), Hispanic-American (HA), Asian-American (AsA)	246 (145 AA, 67 EA, 23 HA, 11 AsA)	rs11730582 rs28357094 rs6532040 rs9138	The C allele of rs9138 polymorphism was associated with photosensitivity (*p* = 0.001, OR 3.245, 95 % CI 1.609–6.542). The C allele of rs11730582 polymorphism was associated with thrombocytopenia (*p* = 0.023, OR 2.12, 95 % CI 1.11–4.04) and hemolytic anemia (*p* = 0.036, OR 2.55, 95 % CI 1.06–6.13)
Kariuki et al. (2009)	African-American (AA), European-American (EA), Hispanic (H)	323/141 (146 AA, 108 EA, 69 H)	rs9138; rs11730582; rs28357094; rs10516800; rs2853749; rs11728697; rs532040; rs1126772; rs7655182	The risk variant of OPN (rs9138C) was associated with higher serum OPN and IFN-α in men (*p* = 0.0062 and *p* = 0.0087, respectively). In women this association was restricted to younger subjects. In AA subjects, rs11730582T and rs28357094G, were associated with anti-RNP antibodies (*p* = 0.0038; OR 2.9, 95 % CI 1.4–6.1 and *p* = 0.021; OR 3.9, 95 % CI 1.1–11.8, respectively)

*SLE* systemic lupus erythematosus, *OPN* osteopontin, *OR* odds ratio, *CI* confidence interval

These data suggest that OPN genetic variants have a key role in creating a background favoring lymphocyte accumulation and leading to the development of autoimmunity. OPN may exert its effect through its capacity to stimulate proliferation and to inhibit apoptosis of lymphocytes or through its capacity to modulate the immune response by inducing Th1 responses and potentiating polyclonal activation of B cells.

Xu et al. (2007a) demonstrated that single nucleotide polymorphism at position 9259 in exon 7 of the *OPN* gene (*OPN* gene 9250C>T) exists in the Chinese Han ethnic population and is associated with SLE. The frequency of TT genotype was significantly lower in SLE patients (*n* = 158) than in the controls (*n* = 180; *p* = 0.001). The frequency of TC genotype was significantly higher in patients compared to controls (*p* = 0.088). When the SLE patients and controls were separated into women and men, significant differences of frequencies were noted in TT genotype, TC genotype and allele in women, but not in men (Xu et al. 2007a). Moreover, this polymorphism appears to be associated with the susceptibility to lupus nephritis in this population. The TT genotype was lower in SLE patients with lupus nephritis (*p* < 0.05) (Xu et al. 2007b). In a large study of 1,141 SLE patients (707 European-American, 434 African-American) Han et al. (2008) performed the population-based case–control analyses. It was reported that minor alleles of rs1126616 and rs9138 (T and C, respectively) were significantly correlated with higher risk of SLE in combined two ethnic groups of males (*p* = 0.0005; OR 1.73, 95 % CI 1.28–2.33) but not in females (Han et al. 2008). Moreover, significant gender–gender interactions in rs1126616 and rs9138 were detected (*p* = 0.001 and *p* = 0.0006, respectively). Furthermore, haplotype analysis identified rs1126616T-rs1126772A-rs9138C which demonstrated association with SLE in general (*p* = 0.02; OR 1.30, 95 % CI 1.08–1.57), especially in males. Han et al. (2008) suggested that *OPN* gene polymorphism is associated with SLE, especially in males and it was the first description of a gender-specific human lupus genetic association. In another study, Trivedi et al. (2011) examined 246 patients diagnosed with SLE, genotyping the rs11730582, rs28357094, rs6532040 and rs9138 SNPs in the *OPN* gene. They proved that photosensitivity in SLE patients was associated with the risk allele rs9138C (*p* = 0.001; OR 3.245, 95 % CI 1.609–6.542). It is known that cutaneous lupus is characterized by photosensitivity, UV-mediated apoptosis of keratinocytes and an inflammatory infiltration into the skin. The accumulation of pDC (IFN-α-producing cells) in the skin was demonstrated to be important in these conditions (Blomberg et al. 2001). In addition, the study of Trivedi et al. (2011) demonstrated that the C allele of rs11730582 polymorphism is associated with thrombocytopenia (*p* = 0.023; OR 2.12, 95 % CI 1.11–4.04) and hemolytic anemia (*p* = 0.036; OR 2.55, 95 % CI 1.06–6.13). Previous study of Kariuki et al. (2009) revealed an association of the rs9138C allele with higher levels of OPN and IFN-α in a small cohort of male SLE patients (*p* = 0.0062 and *p* = 0.0087, respectively). The mechanism by which *OPN* gene polymorphism modulates serum IFN-α is unclear but murine data suggest a role of OPN in IFN-α production by pDC (Shinohara et al. 2006). Moreover, in African-American SLE subjects (*n* = 434), participating in the study of Kariuki et al. (2009), two SNPs: rs11730582 and rs28357094, were associated with anti-ribonucleoprotein (anti-RNP) autoantibodies (*p* = 0.0038; OR 2.9, 95 % CI 1.4–6.1 and *p* = 0.021; OR 3.9, 95 % CI 1.1–11.8, respectively).

## Concluding Remarks

This review has summarized the advances in understanding of the role of OPN in pathogenesis and outcome of SLE. Osteopontin, a pleiotropic protein highly expressed by various cell types such as cells of the immune system, regulates immune response. A large number of publications suggest that OPN participates in the pathogenesis of many autoimmune diseases, including SLE. It has been demonstrated that OPN expression is increased in SLE patients and is associated with some clinical manifestations and levels of activity, but with divergences in the different populations studied. Causes of these differences include probably ethnic and environmental factors (diet, medications, tobacco smoking, ultraviolet light) and still unknown factors. The association of SLE and *OPN* gene polymorphism is still rarely reported. However, most of the results indicate that it might be a good molecular marker for the susceptibility to, and severity of SLE. Further studies should improve our understanding of the disease and will contribute to more precise diagnosis and treatment of SLE.

## Current Challenges and Future Research Directions

There are reports suggesting the role of OPN and *OPN* gene polymorphism in pathogenesis and/or clinical manifestations of SLE. Unfortunately, many of these studies do not meet the current rigorous standards for non-biased large-cohort trials. Future research should focus on selecting the best study group to investigate the role of OPN in SLE. Statistical analysis of the results should take into account many factors, including sex, age, ethnic group, diet, medications and environmental factors. Studies of *OPN* gene polymorphism must take into account the gene–gene, gene–environment interactions and ethnic factors. Currently, little work has been focused on understanding the molecular mechanism whereby OPN exerts its various effects. Moreover, both randomized trials and long-term follow-up studies are needed to show the efficacy of treatment of autoimmune diseases (e.g., SLE) with anti-OPN antibodies.

## Abbreviations

SLE: Systemic lupus erythematosus
OPN: Osteopontin
IL: Interleukin
SIBLING: Small integrin-binding ligand N-linked glycoprotein
DCs: Dendritic cells
NK: Natural killer
TNF: Tumor necrosis factor
IFN: Interferon
pDC: Plasmacytoid DC
Th: T-helper
SLEDAI: SLE disease activity index
